# Habitat-Mediated Facilitation and Counteracting Ecosystem Engineering Interactively Influence Ecosystem Responses to Disturbance

**DOI:** 10.1371/journal.pone.0023229

**Published:** 2011-08-04

**Authors:** Johan S. Eklöf, Tjisse van der Heide, Serena Donadi, Els M. van der Zee, Robert O'Hara, Britas Klemens Eriksson

**Affiliations:** 1 Department of Marine Benthic Ecology and Evolution, Center for Ecology & Evolutionary Studies, Groningen University, Groningen, The Netherlands; 2 Community and Conservation Ecology Group, Center for Ecology & Evolutionary Studies, Groningen University, Groningen, The Netherlands; 3 Animal Ecology Group, Center for Ecology & Evolutionary Studies, Groningen University, Groningen, The Netherlands; 4 Department of Marine Ecology, NIOZ Royal Netherlands Institute for Sea Research, Den Burg, Texel, The Netherlands; 5 Biodiversity and Climate Research Center, Frankfurt, Germany; Dalhousie University, Canada

## Abstract

Recovery of an ecosystem following disturbance can be severely hampered or even shift altogether when a point disturbance exceeds a certain spatial threshold. Such scale-dependent dynamics may be caused by preemptive competition, but may also result from diminished self-facilitation due to weakened ecosystem engineering. Moreover, disturbance can facilitate colonization by engineering species that alter abiotic conditions in ways that exacerbate stress on the original species. Consequently, establishment of such counteracting engineers might *reduce* the spatial threshold for the disturbance, by effectively slowing recovery and increasing the risk for ecosystem shifts to alternative states. We tested these predictions in an intertidal mudflat characterized by a two-state mosaic of *hummocks* (humps exposed during low tide) dominated by the sediment-stabilizing seagrass *Zostera noltii*) and *hollows* (low-tide waterlogged depressions dominated by the bioturbating lugworm *Arenicola marina*). In contrast to expectations, seagrass recolonized both natural and experimental clearings via lateral expansion and seemed unaffected by both clearing size and lugworm addition. Near the end of the growth season, however, an additional disturbance (most likely waterfowl grazing and/or strong hydrodynamics) selectively impacted recolonizing seagrass in the largest (1 m^2^) clearings (regardless of lugworm addition), and in those medium (0.25 m^2^) clearings where lugworms had been added nearly five months earlier. Further analyses showed that the risk for the disturbance increased with hollow size, with a threshold of 0.24 m^2^. Hollows of that size were caused by seagrass removal alone in the largest clearings, and by a weaker seagrass removal effect exacerbated by lugworm bioturbation in the medium clearings. Consequently, a sufficiently large disturbance increased the vulnerability of recolonizing seagrass to additional disturbance by weakening seagrass engineering effects (sediment stabilization). Meanwhile, the counteracting ecosystem engineering (lugworm bioturbation) reduced that threshold size. Therefore, scale-dependent interactions between habitat-mediated facilitation, competition and disturbance seem to maintain the spatial two-state mosaic in this ecosystem.

## Introduction

One of the most studied but also debated issues in ecology is the relative importance of factors affecting how organisms and ecosystems respond to disturbance [1], [2], [3], [4]. One factor which may have a fundamental impact is the size of point disturbances; following a disturbance that exceeds a threshold size, local processes often change, recovery slows down, and communities may even develop into alternative stable states [5]. Such scale-dependent responses have typically been explained by weakened competitive exclusion from surrounding individuals, which increases the chance that previously inferior competitors can recruit into and dominate the center of disturbed areas [5], [6], [7]. For instance, ice-scour in hard bottom rockweed communities can trigger shifts to domination by mussels or fucoid macroalgae, if the disturbance is so large that the “whiplash” from surrounding rockweed cannot exclude competitors [8]. Importantly, such shifts are mediated by large disturbances, but ultimately depend on competition and space preemption.

Theory and observation suggests scale-dependent ecosystem shifts can also be caused by increased abiotic stress on recolonizing individuals, when the removal of an “ecosystem engineering” species simultaneously removes the self-facilitation required for recovery. Such disturbance responses should be most common in abiotically stressed environments, where communities depend on facilitation from ecosystem engineers. These are species that via presence and/or function change abiotic conditions, concentrate resources and/or alleviate local stress, which induces positive organism-environment feedback [9], [10], [11]. For instance, attenuation of waves and currents by submerged vegetation creates sheltered and calm microenvironments necessary for their own recruitment [12]. Importantly, such effects typically *exceed* engineers both spatially and temporally, i.e. extends beyond engineer patch edges and outlive engineers [13]. As a consequence, engineers may facilitate recolonization of conspecifics into areas impacted by small disturbances; a phenomenon known as short-range facilitation [14], [15], [16]. If, however, the disturbed area spatially exceeds the range of the facilitation, increased abiotic stress will prevent recovery; so called long-range inhibition [17]. One example is increased hydrodynamic stress within large gaps in canopy-forming submerged vegetation [18], [19]. Due to lack of recovery, the impacted area can in theory be colonized by other species that tolerate or even benefit from the altered abiotic conditions. However, the ecosystem shift (from a state with the engineer to a state without the engineer) will occur even if competitors do not colonize, because increased stress is the factor preventing recovery.

Neither of these mechanism takes into account that those inferior competitors that may recruit into a disturbed area are often ecosystem engineers that modify abiotic conditions in other ways than the original community. Establishment of such engineers may actually counteract the spatial facilitation from un-impacted individuals in surrounding areas, and *increase* stress on recolonizing members of the original community. There are many examples of how contrasting engineering effects speeds up competitive exclusion of “counteracting” engineers via “habitat-mediated competition”, which contributes to the formation of spatial mosaics of engineered patches [11], [20]. For example, the “trophic group amensalism” hypothesis suggests that bioturbators locally suppress suspension-feeders by smothering them [21], and the “biomechanical warfare” hypothesis that antagonistic, counteracting engineers – e.g. sediment-destabilizing bioturbators and sediment-stabilizing plants – locally exclude each other partly by influencing abiotic conditions in ways that benefits conspecifics but impacts antagonists [22]. Because engineering effects (including habitat-mediated competition) exceed engineers spatially and temporally, we suggest establishment of counteracting engineers may in fact *reduce* the threshold disturbance size associated with increased risk for changes in recovery and shifts to alternative states increases. This is because counteracting engineering should *exacerbates* stress on recolonizing individuals, and thereby slow down recovery.

**Figure 1:**
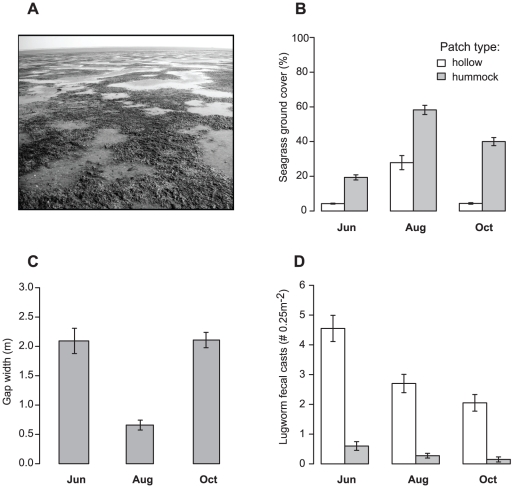
Field survey at the study sites. A) Photograph of site East in June, showing seagrass (*Zostera noltii* L.) patches growing on hummocks next to unvegetated hollows (Photo: Johan S Eklöf), (B) Seagrass ground cover (%) on hummocks and in hollows (n = 40 [20 per site]), (C) average gap width (in m, n = 6 [3 per site]), and (D) lugworm (*Arenicola marina* L.) fecal cast density (0.25 m^−2^) on hummocks and in hollows (n = 40 [20 per site]), in June, August and October. Means ± 1S.E.

We tested these predictions using a field survey and a removal-addition experiment in the eastern Dutch Wadden Sea (Netherlands). The study area was a near-shore intertidal mud flat characterized by a striking mosaic of two alternating habitat types (Fig. 1A): low tide exposed humps (from here on *hummocks*) dominated by the sediment-stabilizing seagrass *Zostera noltii* Hornem (from here on *seagrass*), alternating with low tide water-logged pools or depressions (*hollows*) dominated by the sediment-destabilizing lugworm *Arenicola marina* L. (from here on *lugworms*). Seagrasses can facilitate themselves by stabilizing sediments and reducing erosion and turbidity through attenuation of water flow [12]. In contrast, lugworms are bioturbators that de-stabilize sediments through burrowing and feeding, which in combination with high hydrodynamic activity increases erosion of fine particles [23]. This typically leads to self-facilitation since lugworms prefer sandy substrates [11], but also to competitive exclusion of marine plants which prefer more muddy sediments [22], [24].

After experimentally clearing seagrass and/or adding lugworms from areas ranging in size from 0 to 1 m^2^, we predicted that (i) the strength of seagrass ecosystem engineering and rate of recovery *decreases* with increasing size of disturbance, (ii) lugworm establishment success *increases* with size of disturbance on seagrass due to weakened spatial influence from surrounding seagrass, and (iii) lugworm engineering in disturbed areas *reduces* the threshold disturbance size needed to slow down seagrass recovery by *exacerbating* erosion caused by the removal of seagrass.

## Methods

### Study setting

The study was conducted on the intertidal mudflats at Emmapolder (53°28′ 0 N, 6°45′ 0 E); one of a few areas in the Dutch Wadden Sea where *Zostera noltii* still occurs and has expanded [25]. Growth of this perennial seagrass starts in April, peaks in late summer, and ends in late autumn (October/November) with seasonal senescence [26].

### Field survey

Seagrass (*Zostera noltii*) patches occurred primarily on hummocks, whereas lugworms occurred mainly in gaps (distinct unvegetated areas) which occurred primarily in waterlogged hollows (see Fig. 1A). Since the edges of the patches often extended into the sides of the hollows, gaps were typically somewhat smaller than hollows. To assess the spatiotemporal dynamics of this mosaic, a field survey was conducted in two 100×50 m sites (situated 400 m from the highest shoreline and 350 m apart) in June, August and October 2009. In June, we first measured the relative height of one pair of hummocks and hollows in seven random locations (total n = 14), using a Trimble Spectra Precision LL500 Laser Level (Trimble, California, USA). Measurements were calibrated against two fixed reference metal poles of known height. In all three months, we visually assessed seagrass ground cover to the nearest 5% (a reasonable proxy for shoot density; see Fig. S1), and counted lugworm fecal casts (a good proxy for lugworm density, see Fig. S2) in 0.25 m^2^ frames randomly placed in either of the two habitat types; (i) hummocks and (ii) hollows (n = 20 per habitat type and site, resulting in 240 observations in total). Third, average size of gaps in the seagrass beds were assessed by noting the position of gap edges along 50 m transects randomly placed parallel to the shore (n = 3 transects per site).

#### Statistical analyses

Hypotheses were tested using statistical modeling in the R environment [27]. A one-sided t-test was used to show that the difference in height between hummocks and hollows was greater than zero, after checking assumptions of normality. Effects of Patch type and Time (the three months) on Arc-sin-transformed seagrass cover, and effects of Time on gap width, were assessed using mixed linear models fitted using restricted maximum likelihood (REML) in the nlme package for R. Effects on lugworm densities, which were Poisson-distributed, were investigated using generalized linear mixed modeling with a Poisson distribution and a log-link function (using the *lme4* package). “Time” was treated as fixed (because the levels were chosen to capture seasonality), and “Site” (two levels) was included as random offset, since (i) site effects were not of specific interest but (ii) should be incorporated to reduce within-treatment and reduce the risk for type 1-errors associated with the repeated measures. Test assumptions were first assessed (by inspecting quantile-quantile plots, using the Shapiro-Wilk test for normality, and the Bartlett test for homoscedasticity), and if necessary, data was transformed (square root or log). The full models were fitted down to minimal adequate models using stepwise deletion of non-significant factors (α = 0.05) followed by model fit comparisons using information theory (Aikaike's Information Criterion, or AIC scores).

### Field experiment

A field experiment was conducted between June and November 2009 (the seagrass growth season), testing the relative and interactive effects of size of seagrass clearing (physical removal) and lugworm addition on ecosystem recovery. In each of the two sites (see Field survey), 24 quadratic plots were established within large (>5 m wide) seagrass patches. Plots were placed in a row parallel to the shore, >2 m apart and >2 m from natural gaps to avoid edge effects. Experimental quadratic clearings in plots were conducted in four sizes, mimicking natural disturbances (e.g. waterfowl grazing or uprooting caused by hydrodynamic stress): (i) “Control” (1 m^2^ intact plots), (ii) “Small” (0.0625 m^2^ clearing), (iii) “Medium” (0.25 m^2^ clearing), and (iv) “Large” (1 m^2^ clearing). To experimentally remove only seagrasses (and not the sediments they stabilized), plywood frames were hammered 20 cm deep along plot edges during low water. Next, the frame was filled with water and all seagrass shoots, roots and rhizomes were removed using a hand rake. After allowing suspended particles to settle, the water was slowly released and the frame gently removed. Lugworm (mean g. DW±SE = 0.43±0.05, n = 25) were added on half of the plots (lugworm addition) in densities corresponding to 32 ind. per m^−2^. This relatively high density was intentional, as lugworm densities quickly self-regulate to local carrying capacity due to interspecific competition [28]. All treatment combinations were replicated three times per site.

#### Ecosystem engineering

First, we tested how Clearing size affected seagrass water flow attenuation (after three weeks, to minimize confounding effects of recolonization). This was assessed as % weight loss of plaster dissolution balls; a reliable method when – as here – used in similar water-flow environments [29]. Dissolution balls were molded of model plaster (Knauf Modelgips, Knauf B.V., Utrech, Netherlands) in plastic cups around 20 cm long galvanized steel nails, dried, weighed (mean g. DW±SE = 80.96±2.53, n = 24), and placed in the center of plots for three tidal cycles (40 hrs). No balls were placed in lugworm addition plots, to exclude potential disturbance from nails on added worms. The balls were then retrieved and dried until constant weight. Second, the effects of Clearing size and Lugworm addition on sediment erosion were assessed as the relative gap depth (the difference in height between the center and the mean of two sides of each plot after four and ten weeks), using the Trimble Laser Level; (see Methods: Field survey).

#### Lugworm density and seagrass recolonization rate

Lugworm fecal casts were counted in plots at the start of each month (six times) to assess if (i) the lugworm addition treatment remained over time (high vs. low densities), (ii) seagrass Clearing size, rather than seagrass removal *per se*, facilitated lugworm establishment (see Introduction), and (iii) seagrass presence explained low lugworm densities on natural hummocks (see Results, Field survey). To account for the differences in plot sizes, counts were standardized as no. of casts 0.25 m^−2^; the area sampled in the Field survey. Seagrass recolonization occurred primarily as lateral rhizomal expansion from the edges into gaps (no seedlings were found). Therefore, *relative* rates of recolonization will decrease with clearing size even if the absolute rhizome expansion rate is constant, simply because the distance from the edge to the center increases. However, our “stress-induced shifts” model predicted that *absolute recovery rates* should decrease with increasing clearing size due to weaker engineering effects (see Introduction). We therefore estimated absolute recovery as the average rate at which the quadratic gaps contracted during the foregoing month (in mm day^−1^). This resulted in five monthly estimations of daily gap contraction rates (from start of June until start of November). During days with low water and clear skies, plots were photographed (with a Konica Minolta Dimage ×50 at 5MP resolution) at a 90° angle from 1.5 m height (on the same dates as lugworm counts). The sizes of gaps were estimated to the closest 0.0001 m^2^ using the freeware image analysis program ImageJ [30], by encircling the gap (between the bases of the most inward extending shoots) and measuring the size using a reference marker of known size. We then calculated the “gap contraction rate” (*gcr*, in mm day^−1^) over the foregoing month as:
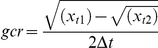
where *x_t1_* and *x_t2_* are gap sizes at start and finish of that month, respectively (in mm^2^), and Δt is the number of days between start (t1) and finish (t2). We also calculated the seagrass relative recovery at the start of each month as “percent of the gap size at start” (in June).

#### Data analyses

Treatment effects were explored using linear mixed effects modeling (see above) with the fixed factors Clearing size, Lugworm addition and Time (after the start of the experiment, where applicable), and their respective interactions, included in full models. “Plot” (n = 48) was included as a random factor, and effects of first order autoregressive and compound symmetry correlation structures were compared to account for effects of repeated measures [31]. “Site” was not included because (i) site differences were not of particular interest, (ii) the low number of levels makes it impossible to assess variation between sites [32], and (iii) potential effects of site differences on variation were accounted for by using individual plots as random offset.

Effects of Clearing size and Lugworm addition on flow-induced weight loss of dissolution balls were tested with a linear mixed effects model with “Site” (two levels) as a random factor (see Methods: Field survey). Effects of Clearing size, Lugworm addition and Time (repeated measure; June and August) on relative gap depth (caused by sediment erosion) were investigated using a mixed-effects model (see Methods: Field survey).

Fecal cast densities (a good proxy for lugworm density) were highly non-normal and zero-inflated. We therefore modeled effects of Clearing size, Lugworm addition, Site and Time using a generalized linear mixed model with a Poisson distribution, using a Bayesian approach. The initial density was modeled as a function of site, clearing size and lugworm addition, with all interactions. Subsequent densities were modeled with additional terms for months, and an autoregressive term for the previous lugworm abundance (i.e. the expected cast density), which varied by month. The time effect and autoregressive terms were given Normal priors with mean 0, and variance 10. The main effects, and first and second order interactions were modeled as random effects, with a Jeffreys prior on the variance (i.e. a uniform prior on the log variance): if interactions are small, this shrinks their effects towards zero [33]: the Jeffrey's prior gives more weight to smaller variances, and these in turn give more weight to small parameter estimates, i.e. estimates that are close to zero. Variances due to the effects were calculated from the distributions of the estimated parameters, rather than from the parameters of the model, as the low number of levels of the factors made the variance estimates uncertain. The fitting was carried out by MCMC in OpenBUGS3 1.2 [34]. Five chains were run, and after a burn-in of 150.000 iterations, a further 150.000 iterations were run, and the chains were thinned to every fifth iteration. Convergence and mixing were judged by eye and with plots of the modified Gelman-Rubin statistic [35].

Finally, effects on seagrass recovery (daily gap contraction rate) were evaluated by fitting a linear mixed effects model with “site” as a random offset, after excluding no clearing (Control) plots (which had no gaps, resulting in a mean and variance = 0).

### Identifying factors(s) predicting the risk for sudden seagrass disturbance

To our surprise, gaps in three treatment combinations expanded on average instead of contracting during October; in Large clearings with and without lugworm addition, and Medium-sized clearings with lugworm addition (see Results). This effect was due to an additional disturbance on the seagrass that had recolonized plots subjected to these initial treatment combinations. Meanwhile, the recolonizing seagrass in other treatment plots were not impacted, and the gaps closed up completely. Even though this unanticipated pattern of additional disturbance was ultimately caused by an interaction between Clearing size and Lugworm addition (see Results), we wanted to in greater detail understand what factor(s) or conditions triggered this disturbance (e.g. the engineers or their effects), and thereby try to identify the additional disturbance. To identify *what local conditions* triggered the additional disturbance, we gathered plot-specific data on five potentially important variables serendipitously collected just before the additional disturbance (early October). The variables were (i) *seagrass gap size* and (ii) *lugworm fecal cast density* (reflecting the two treatments); (iii) *seagrass ground cover* (%) in recolonized areas, which may influence self-facilitation (cover estimated from the plot photographs using standard digitizing [see 36]), (iv) the *elevation* of the area surrounding each plot, (measured in August, see above), which may influence exposure, and (v) the *size of hollows* (water-logged low-tide depressions) encircling the gaps created by the seagrass clearing; a measure of the spatial extent of sediment erosion (estimated from plot photographs, see Methods; Field experiment) (For a summary of all data, see Fig. S3). These variables were then used as predictors in a multiple logistic regression model with “risk for disturbance” as response variable (where 0 = no gap expansion, and 1 = gap expansion, n = 48). “Site” (two levels) was included as a random offset to reduce the influence of natural variation (using the *lmer* function in the lme4 package for R). As “gap size” and “hollow size” were highly correlated (Pearson rho = 0.88), we removed “gap size” (regarded less important because the disturbance occurred outside the gaps but in the hollows). The minimal adequate model was identified as described above.

## Results

### Field survey

In June, hummocks were 5.81±0.5 cm (mean ± SE) higher than hollows (one-sided t-test; t_6_ = 12.41, P<0.001). Seagrass ground cover (Fig. 1B) was at least three times higher on hummocks than in hollows (t = 14.68, P<0.001), increased fourfold between June and August both on hummocks and in hollows, and declined between August and October, but more so in hollows than on hummocks (Habitat×Month interaction; t = 3.7, P = 0.003). Due to the lateral expansion of seagrass into hollows, gaps in the seagrass beds shrunk in width from 2 to 0.6 m between June and August. However, between August and November, gaps expanded back to 2 m in size (t = 7.67, P<0.001). Finally, lugworm cast densities displayed an inverse spatial pattern compared to seagrass cover (Fig. 1D); they were on average eight times higher in hollows than on hummocks (Habitat effect: z = −13.4, P<0.001) and declined throughout the survey (linear Time effect: z = −6.78, P<0.001).

### Experiment

**Figure 2:**
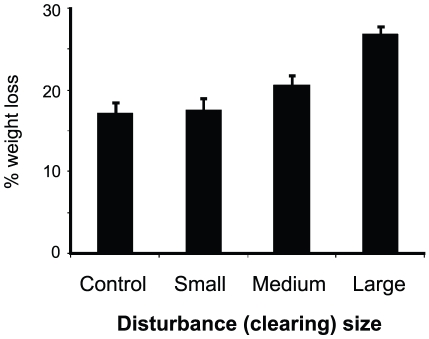
Effect of Clearing size (four levels) on seagrass water flow attenuation (% weight loss of plaster dissolution balls). Means ± 1S.E (n = 6).

**Figure 3:**
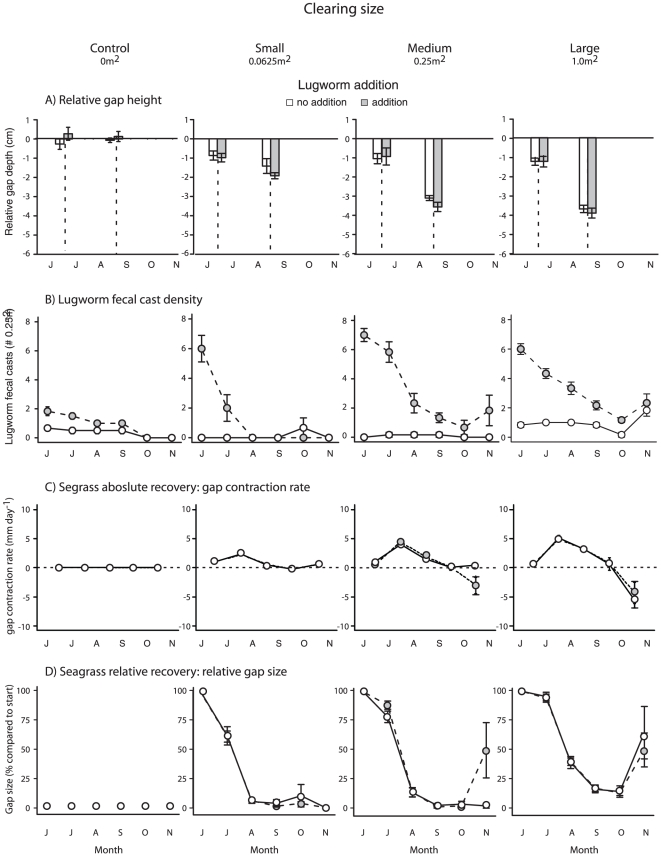
Effects of Clearing size (four levels), Lugworm addition (two levels, white and grey bars/points) and Time (months; Jun–Nov) on four variables reflecting recovery of seagrass ecosystems. Effects on (A) relative gap depth (mm), (B) lugworm (*Arenicola marina*) fecal cast density (0.25 m^−2^), (C) absolute seagrass recovery rate (mm day^−1^), and (D) relative seagrass gap size (% of that at start in June). Means ± 1S.E (n = 6).

Water flow attenuation (measured as % plaster dissolution) and sediment erosion (as relative gap height) indicated that seagrass ecosystem engineering strength in the center of plots depended on the *size* of seagrass clearing, rather than clearing *per se*. Relative weight loss of dissolution balls (Fig. 2) was higher in Large gaps than all other sizes, including no-clearing controls (t = 2.5, P = 0.02). Changes in relative gap depth over time (Fig. 3A) also depended on Clearing size (Clearing size×Time interaction, t = −8.01, P<0.001). The elevation of Control plots did not change between June and August, whereas gaps in clearing plots deepened – especially those in Medium and Large gaps. There was also a trend to an overall deepening of gaps in Lugworm addition plots (t = −1.8, P = 0.079).

Lugworm fecal cast densities were mainly affected by the main effects of Clearing size (explaining 55% of variation), Lugworm addition (20%) and Time (15%); cast densities increased with the size of initial clearing and with lugworm addition, but decreased over time in all treatments (Fig. 3B). The three-way interaction accounted for less than 1% of the total variation (posterior mode 0.5%, 95% highest posterior density interval: 0.03%–3.2%), and the two-way interactions accounted for about 10% of the variation between them (mostly split equally between the interactions involving Clearing size). More worms were found in the largest clearings, 3.7 times more than in the control plot (95% highest posterior density interval: 2.9–5.0). Site had no discernible effect.

The absolute rate of seagrass recovery into the gaps (Fig. 3C) depended on a complex interaction between Clearing size, Lugworm addition and Time (t = −2.72. P = 0.0073). This interaction largely consisted of three effects, all unexpected. First, seagrass recovery rate was between June and September either unaffected by Clearing size, or *higher* in Medium and Large than Small clearings (the opposite to the predicted effect, see hypothesis 1). This was explained by a much faster *relative* recovery in Small than Medium and Large plots in June and July (caused by their smaller size, see Fig. 3D), resulting in that recolonizing seagrass became space-limited in Small, but not in Medium and Large plots, during optimal growth conditions in late July and August (Fig. 3D). Second, lugworm addition did not slow down recovery; instead, lugworms appeared to be outcompeted by the seagrass (Fig. 3B). Third and final; during the last month of the experiment (October), the prevailing recovery trajectories changed drastically in ways that indirectly confirmed our third hypothesis. In Small plots, and in Medium plots without added lugworms, seagrass continued recovering and gaps contracted completely (disappeared). However, in those Medium plots where lugworms had been added five months earlier, the recolonizing seagrasses had disappeared in half of the plots. As a consequence, gaps in this treatment expanded (from an average of 0.0063 to 0.12 m^2^), and absolute recovery rates shifted from positive to negative (Fig. 3C). In Large clearings, recolonizing seagrass were similarly lost in 11 of the 12 plots (92%), regardless of lugworm addition. Consequently, these gaps also expanded (from 0.12 to 0.51 m^2^), and recovery rates shifted from positive to negative (Fig. 3C).

### Factors predicting the risk for sudden seagrass disturbance

**Figure 4:**
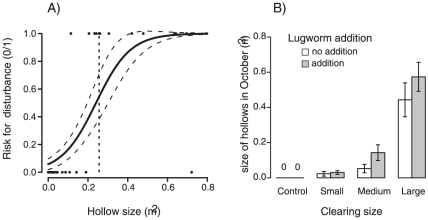
Clearing size and Lugworm addition influences the risk for additional disturbance by influencing hollow size. A) Effect of hollow size (m^2^) in early October on the risk for disturbance on recolonizing seagrass during the following month (0–1). The lines show the predicted effect ± 95% CI (n = 48). The horizontal line depicts the threshold hollow size (0.24 m^2^) where the risk is 50%, (B) Effect of Clearing size and Lugworm addition on October hollow size (m^2^, n = 6). Means ± 1S.E.

The risk for this additional disturbance on the recolonizing seagrass was predicted by hollow size (z = 3.0011, p = 0.0018, Fig. 4B), and was unaffected by seagrass cover, lugworm density and elevation (z = −0.59–0.34, *p*>0.55 for all factors). The threshold hollow size (where disturbance risk = 50%) was ∼0.24 m^2^ (see horizontal line in Fig. 4B). Based on the identification of hollow size as the sole predictor, we assessed how Clearing size and Lugworm addition in June had influenced this variable in early October, by fitting a linear mixed model with “Plot” as random factor, and excluding Control plots (which had no hollows, n = 36). The analysis showed that the size of the hollows was a simultaneous effect of Clearing size (t = 2.92, P = 0.00629) and *Arenicola* addition (t = 2.067, P = 0.046). Hollows in Large plots were 19 and 5 times larger than those in Small and Medium plots, respectively, and Lugworm addition increased hollow size by 45% across clearing sizes (Fig. 4C). Consequently, the risk for additional disturbance and expansion of gaps during October largely depended on the combined scale-dependent effects of seagrass removal and lugworm addition on surface sediment stability.

## Discussion

Spatial mosaics of patches dominated by ecosystem engineers are common in ecosystems in general, and in inter- and sub-tidal marine systems in particular [22], [37], [38], [39], [40], [41]. Such spatial patterning may be explained by local self-facilitation or by simple space preemption [24], but also by habitat-mediated competition where competitors exclude each other by exacerbating local abiotic stress on their competitors [17].

Here, we show that habitat-mediated self-facilitation and habitat-mediated competition may interactively result in scale-dependent ecosystem responses to disturbance. In the largest seagrass clearings (1 m^2^), local environmental conditions shifted from sediment stabilizing to de-stabilizing over the course of the experiment regardless of lugworm addition, most likely because of weakened (i) water flow attenuation and (ii) sediment stabilization (the latter caused by a slower relative recovery rate). This essentially caused the formerly seagrass-dominated low-tide hummocks to switch into low-tide hollows during the autumn. However, this scale-dependent response was not due to simple slowing down of recovery due to lack of facilitation [e.g. 42] or space preemption by competitors [e.g. 8], but greatly increased risk for additional disturbance on recolonizing seagrass. Likewise, lugworm addition did not slow down seagrass recolonization, but lugworm surface sediment destabilization increased the size of hollows surrounding gaps. This was, in turn, the sole factor influencing the risk that recovering seagrass were disturbed again during autumn. As a consequence, the counteracting lugworm engineering decreased the threshold experimental clearing size associated with the additional disturbance from 1 to 0.25 m^2^. Consequently, counteracting engineering reduced the threshold size of disturbance necessary to cause shifts in ecosystem as predicted, but via a mechanism that was largely unexpected.

**Figure 5:**
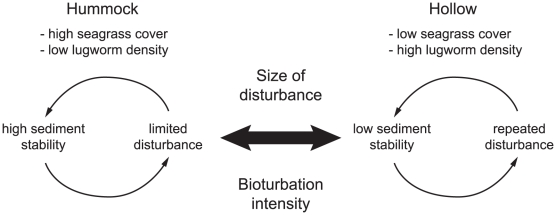
Conceptual model of how seagrass-dominated hummocks and lugworm-dominated hollows could constitute two alternative and potentially stable states in intertidal soft-bottom areas. Local seagrass cover and lugworm density interactively affects sediment net stability, which in turn determines the risk for disturbance on seagrass. The effects of disturbance feeds back positively on local sediment stabilization the following season, so that hollows disturbed one year has an increased risk of being disturbed again the following year. Shifts between the states should be mediated primarily by factors influencing local sediment stability; the size of disturbance on seagrass and lugworm bioturbation intensity, but also by risk for disturbance (e.g. geese grazing intensity or hydrodynamic stress), sediment relative erodability, and seagrass engineering strength.

The scale-dependent seagrass recolonization was – in contrast to our expectations – not caused by slower recolonization, but by increased risk for disturbance on recolonizing seagrasses during autumn. Since the study was not designed to identify the additional disturbance or the underlying cause(s) to it, we can only speculate on which was the disturbance. Visual observations in the field strongly suggested that waterfowl grazing – a well-known seasonal disturbance on *Z. noltii* in the Wadden Sea [26], [43], [44], [45] – was the most likely candidate. Flocks of 150–200 Brent geese (*Branta bernicla*) and Wigeon (*Anas penelope*) were observed feeding daily on *Zostera noltii* from mid September to November (van der Heide et al., in preparation). Importantly, the grazing was spatially restricted to seagrasses in hollows, while seagrass-dominated hummocks covered 50% of the area (van der Heide et al. in preparation). Such hollow-specific waterfowl grazing has been observed elsewhere, and is explained by the low-tide standing water in hollows, which facilitates feeding while reducing ingestion of silt [46]. Moreover, geese typically cease feeding if food presence and/or accessibility is too low (“giving-up density”), which may happen when seagrass still remain [47]. Combined with our results, this suggests the disturbance on the recolonizing seagrass was waterfowl grazing in hollows of a size that harbored enough accessible food. Similar spatial thresholds in grazing have been demonstrated in terrestrial grasslands, and can sustain repeatedly grazed patches of short vegetation next to patches of high, ungrazed vegetation [48]. Until these suspected effects of waterfowl grazing have been thoroughly tested with experiments [see e.g. 43], we do not exclude the possibility that other disturbances (like uprooting due to increased hydrodynamics, see Fig. 2) contributed to or caused the seagrass loss. Regardless of which disturbance caused the seagrass loss, hollow size alone predicted the risk for sudden seagrass disturbance (Fig. 4A). Meanwhile, large enough hollows were caused by the combined effect of large disturbance and lugworm addition (Fig. 4B). Combined with the fact that disturbances on seagrass during autumn are already known to reduce local sediment stabilization in the end of the following growth season [43], this indicates the existence of a positive feedback between disturbance on seagrass and low sediment stability. If strong enough, such a feedback could trigger the formation of two alternative and potentially stable states; (i) undisturbed hummocks with high sediment stability, and (ii) repeatedly disturbed hollows with low sediment stability (Fig. 5). Shifts between the states should – based on our results – be mediated by factors influencing sediment stability; the size of disturbance and the bioturbation by lugworms. However, they may also be influenced by the overall risk for additional disturbance (e.g. geese density or hydrodynamic conditions), the erodability of sediments (e.g. silt content), and the strength of seagrass engineering (e.g. related to shoot density and height). The hypothesis of two alternative states is, moreover, supported by the pervasive spatial hummock-hollow mosaic in our study area (Fig. 1A–D), which is very similar to the bimodal state distribution typical for feedback-driven systems [see e.g. 17], [22]. However, to prove that the states are truly *persistent* [49], it must be demonstrated that the risk for additional disturbance not only depends on hollow size, but that disturbed hollows are much more prone to additional disturbance the following year.

Habitat-mediated competition can alone maintain spatial mosaics if counteracting engineering is strong enough to exclude competitors [11], [20], [22], [50]. In our study area, lugworms were too weak of a competitor to exclude seagrasses. This unexpected result contrasts with documented seagrass exclusion by lugworms in nearby areas [24], and suggests the relative engineering strength of lugworms – and thereby the outcome of habitat-mediated competition – is conditional i.e. depends on other factors (e.g. sediment conditions and hydrodynamic conditions). This may explain the sometimes conflicting evidence for competition between bioturbators and intertidal plants around the world [22], [37], [38], [39], [40], [41]. However, even though lugworms were more or less outcompeted by the end of the summer in our study, their destabilization of sediments increased the size of hollows, which in medium-sized clearings exacerbated the risk for additional disturbance. This demonstrates that bioturbation can exacerbate scale-dependent responses, in the same way as loss of sediment-stabilizing engineers has been shown to slow recovery [42]. It also emphasizes that such engineering effects may locally outlive engineers [13]. Moreover, lugworm densities in fact increased during the last month in plots where the recolonizing seagrasses were disturbed (see Fig. 3B, Medium and Large hollows during October). This suggests that removal of a superior competitor (seagrass) facilitated the starting point of population turnover; another prerequisite for persistence of an alternative state [49].

In conclusion, our study supports the notion of biota as an important factor for disturbance responses and as drivers of ecosystem heterogeneity [5], [17], [51]. The results illustrate that habitat-mediated self-facilitation, counteracting ecosystem engineering and physical disturbances should not be viewed in isolation, because they may interactively explain ecosystem trajectories following disturbance, and, over time, ecosystem spatial heterogeneity. Even though this study appears to be the first demonstrating the potential importance of such interactions, we believe they are a common in engineered systems [see e.g. 22]. Finally, from a local Wadden Sea perspective, the results strengthen the hypothesis [51] that recent shifts from a historical dominance of sediment-stabilizing engineering species like seagrass and reef-forming mussels, to current dominance by bioturbators such as lugworms is caused by species-specific disturbances (e.g. disease, eutrophication, overgrazing, harvest, etc.), but maintained by sediment-mediated positive feedback interactions.

## Supporting Information

Figure S1 Relationship between *Zostera noltii* ground cover and seagrass shoot density, above-ground biomass and total biomass. To test how accurately estimations of *Zostera noltii* ground cover predicted *Z. noltii* shoot density and biomass, we in the field placed a 0.25 m^2^ frame in random points ranging in seagrass cover from 5 to 90% (n = 6), photographed the frame (see the article for details), and excavated all seagrass material in the center of the frame using a 10.5 cm wide corer (to 20 cm depth). Seagrass ground cover in each frame was estimated with the same digital photography analysis as in the experiment (see article and Stewart et al. 2007). In the laboratory, we counted all shoots, separated and cleaned the above- and below-ground material in seawater, and dried both fractions until constant dry weight (at 60°C). Results from multiple regressions showed that estimated cover was a reasonable predictor of shoot density (F_1,5_ = 14.4, P = 0.012, R^2^ = 0.69), above-ground biomass (F_1,5_ = 21.3, P = 0.0056, R^2^ = 0.77) and total biomass (F_1,5_ = 20.47, P = 0.0063, R^2^ = 0.76).
(EPS)

Figure S2 Relationship between *Arenicola marina* density and lugworm fecal cast density. Counts of *Arenicola marina* fecal casts is a commonly used and rapid method to assess worm densities (see e.g. van Weseenbeck et al. 2007 in article reference list). To ensure the reliability of the method in our study area, we compared counts of fecal casts in randomly placed 0.25 m^2^ frames (both within and outside of *Zostera noltii* patches) with actual densities of worms in the sediment, by excavating all worms to 50 cm depth within the frame (n = 10). A linear regression analysis showed that the number of fecal casts accurately predicted worm densities (F_1,8_ = 67.00, P<0.0001, R^2^ = 0.88).
(EPS)

Figure S3 A–D. Five predictor variables used in multiple logistic regression analysis, as effects of Clearing and Lugworm addition (across two sites, means ± 1SE, n = 6).
(EPS)
